# Acid-Resisting Mackintosh

**Published:** 1911-09-09

**Authors:** 


*To the Editor of* The Hospital.

### ACID-RESISTING MACKINTOSH.

*To the Editor of* The Hospital.

Sir,—Can you or any of your readers aid me in procuring a mackintosh for a bad bedridden case? I have tried several kinds, with little success. I have two squares of mackintosh going at a time, so that when one is under the patient the one removed can be laid for a few minutes in Jeyes' Fluid (diluted) and hung out in the open air to dry and sweeten, and yet with all this careful attention the mackintoshes only last about two months. The mackintosh I am using now is "special Wigan," at 5s. 9d. per yard. Is there a special make made for such cases that will stand against uric acid, etc., and if so, where can it be procured?—I am, yours truly,

